# Nutritional Improvement of Gluten-Free Breadsticks by Olive Cake Addition and Sourdough Fermentation: How Texture, Sensory, and Aromatic Profile Were Affected?

**DOI:** 10.3389/fnut.2022.830932

**Published:** 2022-02-10

**Authors:** Giusy Rita Caponio, Graziana Difonzo, Giuditta de Gennaro, Maria Calasso, Maria De Angelis, Antonella Pasqualone

**Affiliations:** ^1^Department of Soil, Plant and Food Sciences, University of Bari Aldo Moro, Bari, Italy; ^2^Clinica Medica “A. Murri”, Department of Biomedical Sciences and Human Oncology, University of Bari Medical School, Bari, Italy

**Keywords:** gluten-free, olive cake, glycemic index, texture, upcycling, *Lactiplantibacillus plantarum*, *Leuconostoc mesenteroides*

## Abstract

There is a growing need for gluten-free bakery products with an improved nutritional profile. Currently, gluten-free baked goods deliver low protein, fiber, and mineral content and elevated predicted glycaemic index (pGI). Olive cake (OC), a by-product from virgin olive oil extraction, is an excellent natural source of unsaturated fatty acids, dietary fiber and bioactive molecules, including polyphenols and tocopherols. In this framework, this study aimed at using two selected lactic acid bacteria and a yeast for increasing the antioxidant features and the phenol profile of the gluten-free breadsticks fortified with OC with the perspective of producing a functional food. Control (CTR) samples were prepared and compared with fermented ones (fCTR). Samples were added with either non-fermented OC (nfOC) or fermented for 12 and 20 h (fOC-12 and fOC-20). Our results showed that the predicted glycemic index (pGI) was influenced by both OC addition and sourdough fermentation. In fact, the lowest value of pGI was found in fOC-12, and hydrolysis index and pGI values of samples with OC (fOC-12 and nfOC) were statistically lower than fCTR. Both OC addition and fermentation improved the total phenol content and antioxidant activity of breadsticks. The most pronounced increase in hardness values was observed in the samples subjected to sourdough fermentation as evidenced both from texture profile analysis and sensory evaluation. Moreover, in most cases, the concentration of the detected volatile compounds was reduced by fermentation. Our work highlights the potential of OC to be upcycled in combination with fermentation to produce gluten-free breadsticks with improved nutritional profile, although additional trials are required to enhance textural and sensory profile.

## Introduction

Celiac disease is a global health problem with ~1% of the world population being affected (1, 2). Moreover, gluten also causes other pathologies grouped under the term “gluten-related disorders,” namely, non-celiac gluten sensitivity, dermatitis herpetiformis, gluten ataxia and wheat allergy (3).

A gluten-free (GF) diet requires the complete exclusion of gluten, a protein complex present in food products from wheat, rye, barley, oats, spelt, kamut or their hybrids. It only comprises naturally GF food products (e.g., legumes, fruit and vegetables, unprocessed meat, fish, eggs, and dairy products) and/or substitutes of wheat-based foods, specially manufactured without gluten or having a gluten content lower than 20 mg/kg, according to the Commission Regulation (EC) No 41/2009 (4).

There are different challenges to face in the production of GF bakery products. First of all, gluten is responsible for several important technological features, and it is a big task to find ingredients that are able to mimic its properties and develop sensory acceptable GF baked goods. Moreover, the nutritional intake given by GF products must also be considered. Frequently, the celiac diet is unbalanced, with a high intake of saturated fatty acids and sugars and lacking in different nutrients, such as dietary fiber, iron, zinc, magnesium, calcium, B12 vitamin, and folate (5–7). Furthermore, GF products often present a high predicted glycaemic index (pGI) due to their starch-based composition. A high pGI represents a problem for subjects affected by metabolic disorders, such as obesity and diabetes (8).

The olive mill generates large amounts of different by-products, such as olive leaves, olive pomace, olive mill wastewaters and olive stones. These by-products have attracted attention for their possible exploitation in several industrial sectors (9, 10). The recently introduced multi-phase decanter technology for olive oil industrial extraction generates large quantities of olive cake (OC), a novel by-product made up of the partially defatted wet drupe pulp that contains very low traces of the stones. Recently, different authors reported that OC is an excellent natural source of unsaturated fatty acids, dietary fiber and biologically active substances, including polyphenols, triterpenic acids, tocochromanols and carotenoids (11, 12).

Different processing techniques may enhance the biological value of by-products by eliminating or reducing some technological drawbacks, such as unappealing taste, when used in baked goods. Sourdough fermentation, also in combination with enzymes, is one of the best tools for the above purposes (13). Bioconversion processes and fermentation strategies of agri-food by-products can increase digestibility, enhance nutritional value and decrease the levels of anti-nutritional factors in these substrates. Microbial fermentation of agri-food waste is used to produce ingredients with improved nutritional and health attributes, favoring the release of bioactives from the vegetable matrix, or through bioconversion of the compounds originally encountered in the by-products (14). For instance, fermentation of olive-mill wastewaters by *Yarrowia lipolytica* to produce citric and oleic acid has been reported (15). Specific compounds produced through microbial fermentation are largely dependent on the raw material characteristics and the specific microbial species used. Innovative valorization strategies include bioconversion of vegetables by-products using lactic acid bacteria (LAB) and other microorganisms to obtain enzymes, polysaccharides, nutraceuticals and beverages (16). Strategies based on sourdough fermentation to elaborate baked goods, which may ameliorate the symptoms of irritable bowel syndrome and with enhanced nutritional value, have been described (13, 17). The wide microbial biodiversity holds a great array of metabolic potential that is useful to design fermentation strategies and obtain added-value ingredients through vegetable residue fermentation, which would have the greatest environmental implication because of the large volume involved (14).

Based on those outcomes, our aim was to improve some nutritional features of GF breadsticks by upcycling OC and taking advantage of sourdough fermentation. Moreover, the effect on texture, nutritional, sensory and aromatic profile of breadsticks was evaluated.

## Materials and Methods

### Materials

Rice and cornflour, water, sunflower oil, salt and baking powder for the production of GF breadsticks were purchased from local retailers. *Psyllium* fiber and chicory inulin (COSURA groupe Warcoing S.A, Warcong, Belgium) were used in the formulation. OC, purchased from a local olive mill (Oleificio Ferrara, Trani, BAT), was lyophilized (Buchi Lyovapor^TM^ L-200, Switzerland) at −58°C, 0.8 mbar, until it reached 2–3% of moisture, then ground by a laboratory mill and stored in a powder form at −20°C until use.

### Microbial Strains and Culture Conditions

*Hanseniaspora uvarum* CNG11, *Kluyveromyces marxianus* KL, *Lactiplantibacillus plantarum* V3-D0001, *L. plantarum/Pediococcus pentosaceus* CBD 100-D0001, *Leuconostoc mesenteroides* DDL1, *Lc. mesenteroides* KI6, *Pichia kudriavzevii* DCNa1, *Saccharomyces cerevisiae* DDNd10 and *Saccharomyces subpellicelosus* DFNb1 were used.

*Lc. mesenteroides* DDL1, *P. kudriavzevii* DCNa1, *S. cerevisiae* DDNd10, *S. subpellicelosus* DFNb1 (all isolated from date), *Lc. mesenteroides* KI6 (isolated from kiwi) and *Hanseniaspora uvarum* CNG11 (isolated from dogwood) strains belonging to the culture collection of the Department of Soil, Plant and Food Science of the University of Bari, Italy, were previously isolated and identified by partial sequencing of the 16S rRNA (18, 19). *L. plantarum* V3D0001, the mixed *L. plantarum*/*P. pentosaceus* CBD 100-D0001 and *K. marxianus* KL were commercial active dried strains (Sacco System s.r.l., Cadorago, Como, Italy) selected for their established role of vegetable starter cultures. Stock cultures were at −25°C, with 200 μl ml^−1^ glycerol as a cryoprotective agent. For the activation of the cultures, all strains were thawed at room temperature and then LAB strains were routinely cultivated at 1% (v/v) in De Man, Rogosa and Sharpe (MRS) (Oxoid, Basingstoke, Hampshire, England) broth at 30°C for 48 h. Yeasts were routinely propagated at 25°C on Yeast Extract Peptone Dextrose (YPD) broth, containing 10 g of glucose, 5 g of peptone, 3 g of yeast extract and 3 g of malt extract per liter (all ingredients were purchased by Oxoid).

### Strains Selection for Olive-Cake Fermentation

The MRS or YPD broth with increasing OC concentration were used to evaluate the potential inhibitory effect of the OC extracts. OC was dissolved in saline (NaCl 9 g L^−1^) and added to MRS or YPD in a concentration series ranging from 1 to 20 g L^−1^, chosen based on both of the data reported in the literature and deriving from growth preliminary trials. In all cases, the OC solution was sterilized through membrane filtration using Millex^®^ Syringe Filter Units (pore size, 0.22 μm; Merck, Millipore, Darmstadt, Germany) and added aseptically to the sterile MRS and YPD medium. An MRS and YPD broth without the addition of OC extract were used as control. Each LAB and yeast strain was cultivated two times in conventional MRS and YPD broth at 30 and 25°C until stationary growth phase. Cells were harvested by centrifugation at 10,000 × g for 10 min at 4°C, washed twice with 20 mM sterile potassium phosphate buffer (pH 7.0), and re-suspended in distilled water to a final cell density of 9 log colony forming unit (CFU) ml^−1^. Aliquots of each microbial suspension (100 μl) were singly used to inoculate 10 ml of the culture medium containing OC at a final cell density of ca. 7 log CFU ml^−1^. The incubation was at 30 and 25°C for LAB and yeast, respectively. After 24 h, cell density was estimated by pour-plating in MRS and YPD agar and incubated at 30 and 25°C for 48 h. The pH was estimated by a pH meter (Denver Instrument, USA). Three separate experiments were performed, and the average value and standard deviation were calculated. *L. plantarum, L. plantarum*/ *P. pentosaceus* and *K. marxianus* were the most growing and acidifying strains and were selected and used as mixed starters for dough fermentation.

### Dough and Gluten-Free Breadstick Preparation

**Figure 1:**
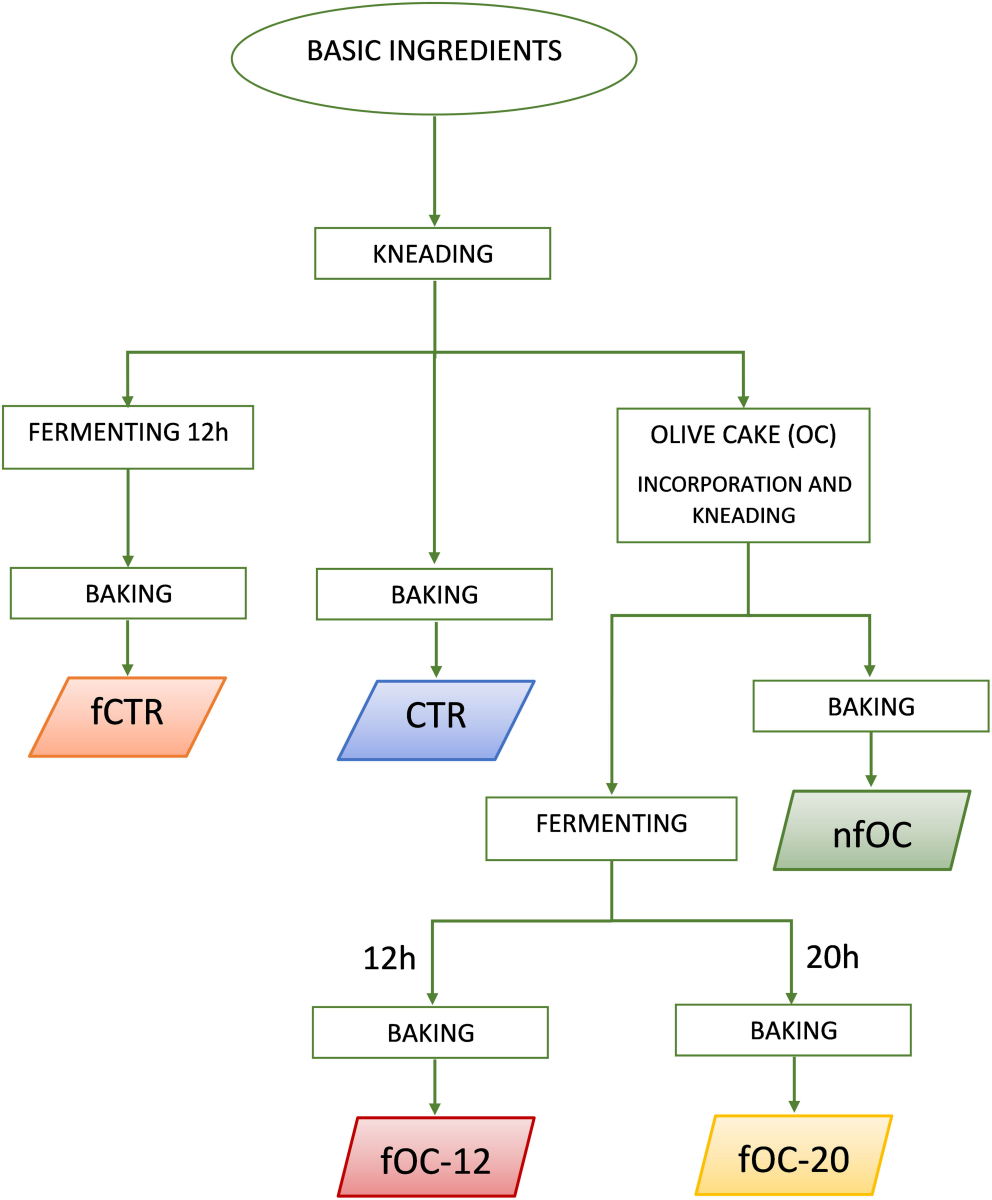
Flowchart of the breadstick sampling. CTR, control breadsticks obtained by the dough without olive cake (OC) addition and without microbial fermentation; fCTR, breadsticks obtained by the dough without OC addition and fermented; nfOC, breadsticks obtained by the dough supplementation with 1% OC without microbial fermentation; fOC-12, breadsticks obtained by the dough supplementation with 1% OC and fermented for 12 h; fOC-20, breadsticks obtained by the dough supplementation with 1% OC and fermented for 20 h.

Gluten-free (GF) breadstick preparation was performed in a pilot plant belonging to the Department of Soil, Plant and Food Sciences of the University of Bari (Italy) on 3 consecutive days (a total of 3 batches for each breadsticks variant). Tap water (37 g 100 g^−1^ dough) at room temperature was mixed with oil (9 g 100 g^−1^ dough). Then, inulin (1 g 100 g^−1^ dough) and *psyllium* fiber (1 g 100 g^−1^ dough) were added and dissolved by gently stirring to obtain a gel. Rice flour (41 g 100 g^−1^ dough), cornflour (9 g 100 g^−1^ dough), salt (0.5 g 100 g^−1^ dough) and baking powder (1.5 g 100 g^−1^ dough) were added to the gel. The ingredients were kneaded for 10 min to obtain the dough. Figure 1 reports the flowchart of the sampling. Different types of GF breadsticks (Table 1) were produced as follows: control breadsticks (CTR) obtained by the dough without OC addition and without microbial fermentation; breadsticks obtained by the dough without OC addition and fermented by *L. plantarum, L. plantarum*/*P. pentosaceus* and *K. marxianus* (fCTR); breadsticks obtained by the dough supplemented with 1% OC without microbial fermentation (nfOC); and breadsticks obtained by the dough supplemented with 1% OC and fermented by *L. plantarum, L. plantarum*/*P. pentosaceus* and *K. marxianus* (fOC). To determine the best fermentation time, both fCTR and fOC doughs were inoculated by the selected mixed starter and fermented at 30°C for 24 h. The pH decrease was then monitored. The selection of fermentation parameters was based on the time to reach the 4.6 pH value. OC was added in substitution of an equal amount of cornflour; thus, the amount of dry ingredients and water was the same in the control and in the experimental doughs. For fermented doughs, overnight cultures of the selected strains (cell count of ~9.0 ± 0.2 log CFU ml^−1^) were harvested by centrifugation (10,000×g, 10 min, 4°C) and washed twice with sterile 20 mM potassium phosphate buffer, pH 7.0. Then, each cell pellets were re-suspended in 1 ml of tap water needed for making the dough and added at an initial cell density of ca. 7.0 and 5.0 log CFU g^−1^ for LAB and yeasts, respectively. The resulting doughs (immediately or at the end of fermentation) were manually shaped to form the breadsticks (9 × 1 cm), which were boiled in water for 3 min and then dried for 5 min on a clean cloth. The boiled breadsticks were baked in a forced-air convention oven at 200°C for 26 min. Baked samples were immediately subjected to the direct analyses and/or to the extraction process. At the beginning and at the end of fermentation, pH was recorded.

**Table 1:** Ingredients used for the gluten-free breadstick formulation.

**Ingredients**	**CTR**	**fCTR**	**nfOC**	**fOC-12**	**fOC-20**
Rice flour (g 100 g^−1^)	41	41	41	41	41
Corn flour (g 100 g^−1^)	9	9	8	8	8
Olive pomace (g 100 g^−1^)	–	–	1	1	1
Inuline (g 100 g^−1^)	1	1	1	1	1
*Psyllium* (g 100 g^−1^)	1	1	1	1	1
Tap water (g 100 g^−1^)	37	37	37	37	37
Sunflower oil (g 100 g^−1^)	9	9	9	9	9
Powdered chemical yeast (g 100 g^−1^)	1.5	1.5	1.5	1.5	1.5
Salt (g 100 g^−1^)	0.5	0.5	0.5	0.5	0.5
Microbial inoculum (log CFU g^−1^)	–	7	–	7	7

*CTR, control breadsticks obtained by the dough without olive cake (OC) addition and without microbial fermentation; fCTR, breadsticks obtained by the dough without OC addition and fermented; nfOC, breadsticks obtained by the dough supplementation with 1% OC without microbial fermentation; fOC-12, breadsticks obtained by the dough supplementation with 1% OC and fermented for 12 h; fOC-20, breadsticks obtained by the dough supplementation with 1% OC and fermented for 20 h*.

### Proximate Composition of Olive Cake and Breadsticks

The lipids, proteins (total nitrogen × 6.25), ash and total dietary fibers were determined according to Association of Official Analytical Collaboration (AOAC) methods 945.38F, 979.09, 923.03 and 991.43, respectively. Moisture content was determined by an automatic moisture analyser at 105°C (Mod. MAC 110/NP, Rodwang Wagi Elektroniczne, Poland). The carbohydrate content was determined as difference: 100 – (moisture + proteins + lipids + ash). The determinations were carried out in triplicate.

### Enumeration of LAB and Yeasts Cell Densities

Lactic acid bacteria (LAB) and yeast cell densities were determined according to methods previously described (20, 21), using culture media and supplements purchased from Oxoid. For each sample, 10 g were diluted with 90 ml of 9 g L^−1^ NaCl solution, homogenized with a Stomacher Lab-Blender 400 (Seward Medical, London, United Kingdom) for 3 min, and serially diluted and plated in triplicate in selective media. LAB was enumerated using MRS agar plates with cycloheximide (0.1 g L^−1^) at 30°C for 48 h under anaerobiosis (AnaeroGen and AnaeroJar, Oxoid). The number of yeast cells was estimated at 25°C by using YPD agar supplemented with chloramphenicol (0.1 g L^−1^) for 48 h. The microbiological counts were confirmed by taking representative colonies for each medium, which were analyzed for morphology, motility, Gram staining reaction and catalase test.

### Chemical Characterization and Antioxidant Activity

The chemical composition and the antioxidant profile of olive cake (antioxidant activity, total phenols and tocopherols) were performed as reported in Difonzo et al. (12). The determination of total phenols was carried out according to Caponio et al. (22) with some modifications. Two g of ground breadsticks were added to 2 ml of hexane, to remove the oil phase, and 10 ml of methanol:water (80:20 v/v). The samples were stirred with a vortex at 2,000 rpm for 15 min, then sonicated (Ultrasonic cleaner CP104, EIA) for 15 min and centrifugated at 10.000 g min^−1^ for 10 min at 24°C (Thermo Fisher Scientific, Osterodeam Harz, Germany). The hydroalcoholic extract was collected, while the pellet was subjected to a second extraction phase following the same procedure. The hydroalcoholic extracts were filtered through a nylon filter (pore size 0.45 mm, Sigma, Ireland). Total phenols were determined according to the Folin-Ciocalteu assay. Twenty microliters of extract and 100 μl of Folin-Ciocalteu reagent were added to 980 μl of Milli-Q water. After 3 min, 800 μl of Na_2_CO_3_ were added and then incubated for 1 h. The absorbance was read at 720 nm and the results were expressed as mg of gallic acid equivalents per g of breadsticks. Antioxidant activity was determined by 2,2-diphenyl-1-picrylhydrazyl (DPPH) assay as reported by Difonzo et al. (23). Specifically, 50 μl of each extract was added to 950 μl of DPPH solution and incubated for 30 min in the dark. The absorbance was read at 517 nm and the results expressed as μmol Trolox equivalent (TE) g^−1^ dry weight. The determinations were carried out in triplicate.

### *In vitro* Starch Hydrolysis

*In vitro* starch hydrolysis was determined as previously described (24). The procedure mimicked the *in vivo* digestion of starch. Aliquots of doughs, containing 1 g of starch (determined in dough), were subjected to enzymatic process (pancreatic amylase and pepsin-HCl), and the released glucose content was measured with D-Fructose/D-Glucose Assay Kit (Megazyme, Wicklow, Ireland). Simulated digests were dialyzed (cut-off of the membrane: 12,400 Da) for 180 min. Aliquots of dialysate, containing free glucose and partially hydrolysed starch, were sampled every 30 min and further treated with amyloglucosidase. Then, free glucose was determined using the above-mentioned enzyme-based kit and finally converted into hydrolysed (digested) starch in the dough. Control white wheat bread was used as the control to estimate the hydrolysis index (HI = 100). The pGI was calculated using the equation: pGI = 0.549 × HI + 39.71 (25). Each sample was analyzed in triplicate.

### Color Determination

The breadsticks color was determined by Chromameter CM-600d (Konica Minolta Sensing, Osaka, Japan). The color indices analyzed for breadsticks were lightness (L^*^), redness (a^*^), and yellowness (b^*^). The analyses were carried out in triplicate.

### Textural Properties

Textural properties of GF breadsticks were determined through a three-point bending test. A Z1.0 TN (Zwick/Roell, Ulm, Germany) was used, equipped with 1 KN load cell. The distance between the support bars was set at 60 mm, and the breadsticks were placed centrally with the probe speed set at 3 mm s^−1^. The maximum force required to break the breadstick (N) and the distance before the breadstick broke (mm) were measured, which represent the hardness and brittleness, respectively. The analyses were carried out in triplicate.

### Sensory Analysis

The sensory analysis was performed by a semi-trained panel of seven panelists that included women and men. Ethical guidelines of the laboratory of Food Science and Technology of the Department of Soil, Plant, and Food Science-University of Bari (Italy) were followed. Panelists were usual consumers of the food category under the study who underwent 1 h training on the definition of the attributes *via* physical references; information about study aims were given, and individually written informed consent was obtained from each participant. The five GF breadsticks were randomly coded with a three-digit number and analyzed on the same day of production. Nine descriptors were chosen: 2 for visual analysis (color intensity, surface homogeneity), 2 for retro nasal analysis (malty and olive), 2 for texture attributes (hardness, crispness) and 3 for taste attributes (bitterness, astringency and oily). The perception intensity of the descriptors was assessed on a scale ranging from 0 (no perception) to 10 (maximum intensity).

### Volatile Profile

Volatile compounds of GF breadsticks were analyzed by a headspace solid phase micro-extraction (HS-SPME) coupled with gas-chromatography/mass spectrometry (GC-MS) following the procedure reported by Difonzo et al. (26). In particular, 0.5 g of each sample were added with 4 ml of a saturated aqueous solution of NaCl and 150 μl of 1-propanol (internal standard). Vials were homogenized using a laboratory vortex for 2 min. The extraction of volatile compounds was performed by exposing a 75 μm Carboxen/polydimethylsiloxane (CAR/PDMS) SPME fiber (Supelco, Bellefonte, PA, USA) in the headspace of the vials containing the samples at 50°C for 40 min. The fiber was desorbed for 6 min in the injection port of the gas-chromatography operating a split-less mode at 230°C for 3.5 min. The determination was carried out using an Agilent 6580 gas-chromatography equipped with an Agilent 5975 mass-spectrometer (Agilent Technologies Inc., Santa Clara, CA, USA). Volatile compounds were separated with a capillary column HP-Innowax (60 m length × 0.25 mm i.d. × 0.25 μm film thickness) under the following conditions: injector temperature of 250°C, oven temperature was held at 35°C for 5 min, then increased to 210°C at 5.5°C min^−1^. This temperature was kept constant for 5 min. Mass detector was set at the following conditions: interface temperature of 230°C, source temperature of 230°C, ionization energy of 70 eV, scan range of 33–260 amu. Helium was used as a carrier with a pressure 30 kPa and a flow of 1 ml min^−1^. Volatile compounds were identified comparing the peak areas of compounds of interest with the area of internal standard. The analyses were carried out in triplicate.

### Statistical Analysis

Significant differences between the values of all parameters were determined at *p* < 0.05, according to the analysis of variance (ANOVA) followed by the Tukey's Honestly Significant Difference (HSD) test for multiple comparisons. The statistical analysis was performed by the Minitab Statistical Software (Minitab Inc., State College, PA, USA).

## Results and Discussion

### Olive Cake Characterization

The proximate composition of the freeze-dried OC, as well as the antioxidant activity in terms of phenolic compounds and tocopherols is detailed in Table 2. The final moisture of the OC was about 3 g 100 g^−1^, with a protein content of 7.47 g 100 g^−1^ and ashes of 9.43 g 100 g^−1^. The lipid fraction is a mixture of saturated (SFA), monounsaturated (MUFA) and polyunsaturated fatty acids (PUFA); MUFA represented the main fraction with a content of 16.66 g 100 g^−1^ of dried cake. In fact, the fatty acid composition resulted that the oleic acid was the most abundant, representing 75% of the total fatty acids followed by palmitic, linoleic and stearic acids. The other fatty acids were found in small quantities (data not shown). These results agree with the literature for olive products and by-products (27, 28). The PUFA/SFA and oleic/linoleic acid (O/L) ratios were 0.72 and 7.89, respectively, in line with those of extra virgin olive oil. The PUFA/SFA ratio is one of the main parameters used to assess the nutritional quality of the lipid fraction of foods. Nutritional guidelines recommend a PUFA/SFA ratio higher than 0.4 (28). In addition, values of O/L equal or higher than 7 indicate a good oxidative stability.

**Table 2:** Chemical composition of the freeze-dried olive cake (g 100 g^−1^ d.m.).

**Proximate composition**
Moisture	2.91 ± 0.05
Fats	22.12 ± 0.76
SFA	3.11 ± 0.12
PUFA	2.24 ± 0.01
MUFA	16.66 ± 0.10
PUFA/SFA	0.72
O/L	7.89
Proteins	7.47 ± 0.16
Ashes	9.43 ± 0.66
Total dietary fiber	20.10 ± 0.62
SDF	4.7 ± 0.08
IDF	15.4 ± 0.12
Carbohydrates (g 100 g^−1^ d.m.)	58.07
**Phenolic content and Antioxidant activity**	
TPC (mg GAE g^−1^)	75.76 ± 0.24
DPPH (μmol TE g^−1^)	274.21 ± 10.72
ABTS (μmol TE g^−1^)	335.20 ± 7.29
Tocopherols (μg g^−1^)	401.21 ± 3.12

*Means value ± standard deviation is reported. SFA, saturated fatty acids; MUFA, monounsaturated fatty acids; PUFA, polyunsaturated fatty acids; O, oleic acid; L, linoleic acid; SDF, soluble dietary fiber; IDF, insoluble dietary fiber; TPC, total phenol content*.

Olive cake (OC) is also a good source of functional molecules, such as dietary fiber, polyphenols, tocopherols and phytosterols, as shown in Table 2. Moreover, OC showed an interesting, though expected, antioxidant activity, especially due to the presence of polyphenols and tocopherols (12, 29).

### Strains Selection for Olive-Cake Fermentation

Due to the increasing need for GF bakery products, food processing methods that improve the nutritional profile and enhance the bioactive compounds are a crucial research topic. As already reported (30–32), it is possible to improve the nutritional properties of OC through fermentation carried out by microorganisms. Sourdough fermentation by selected microorganisms enhances bioactive capacity and improves the technological traits of baked goods (33). To select a suitable microbial strain for OC sourdough fermentation, in the present study, several LAB and yeast strains were screened based on their acidification and growing capacity in OC-added medium after 24 h of fermentation. The OC concentrations that were used in the formulations of the cultivation media (1, 2, 5, 10 and 20 g L^−1^) were chosen considering some preliminary experiments (data not shown) and literature data. Nanditha and Prabhasankar (34) used the synthetic antioxidant butylated hydroxy anisole (BHA) in a range of concentrations between 250 and 2500 ppm, obtaining the inhibition of bacterial and yeast growth (34). Overall, the tested strains, previously recognized as vegetables microbial starter culture or isolated from vegetable source, were able to grow and acidify on an OC-added medium although with different extent (Supplementary Table S1). Maximal growth occurred in broth containing 1 g L^−1^ OC with average increases in cell density of 2.12 log units and an average decrease of pH of 1.60 delta pH units, starting from a pH value of 6.2. No microbial growth was observed when 20 g L^−1^ of OC was added. The highest OC concentration, which allows microbial growth in the tested range, was 10 g L^−1^. In this condition, after 24 h of incubation, the cell density increase was between 0 and 1.59 log units and the pH decrease was between 0.14 and 1.74 pH units (average values). Among the tested strains, the best tolerance was shown by commercial *L. plantarum, L. plantarum/P. pentosaceus* and *K. marxianus* cultures, which showed that the higher cell density increased (1.50 ± 0.01, 1.55 ± 0.05 and 1.59 ± 0.11 log units, respectively) and pH decreased (1.74 ± 0.02, 1.65 ± 0.04 and 0.79 ± 0.11 pH units, respectively) (Supplementary Table S1). The reduced growth, compared to positive control media, can be attributed to the antimicrobial activities of OC extracts related to their content of phenols which have effective antimicrobial activities against a wide array of microorganisms (35), as pointed out by the relevant antiradical activity observed in the extracts (36). OC extracts were rich in phenolic compounds; therefore, phenol tolerance is an important selection criterion for a microorganism to be used as a starter. LAB, mainly belonging to *Lactiplantibacillus, Leuconostoc* and *Pediococcus* genera, has to be adapted to the characteristics of the plant raw materials where phenolic compounds are abundant. In addition, *L. plantarum* is the commercial starter most frequently used in the fermentation of food products of plant origin (21, 22, 37). *K. marxianus* has been shown to be able to grow in olive pomace by solid-state fermentation and improve its nutritive value by simultaneous production of gallic acid (38). In a recent work (39), olive mill wastewater and olive paste were used to improve the chemical quality of bread and pasta. The fermented olive paste was also used to increase the functionality of Italian bakery products *taralli* (11).

**Figure 2:**
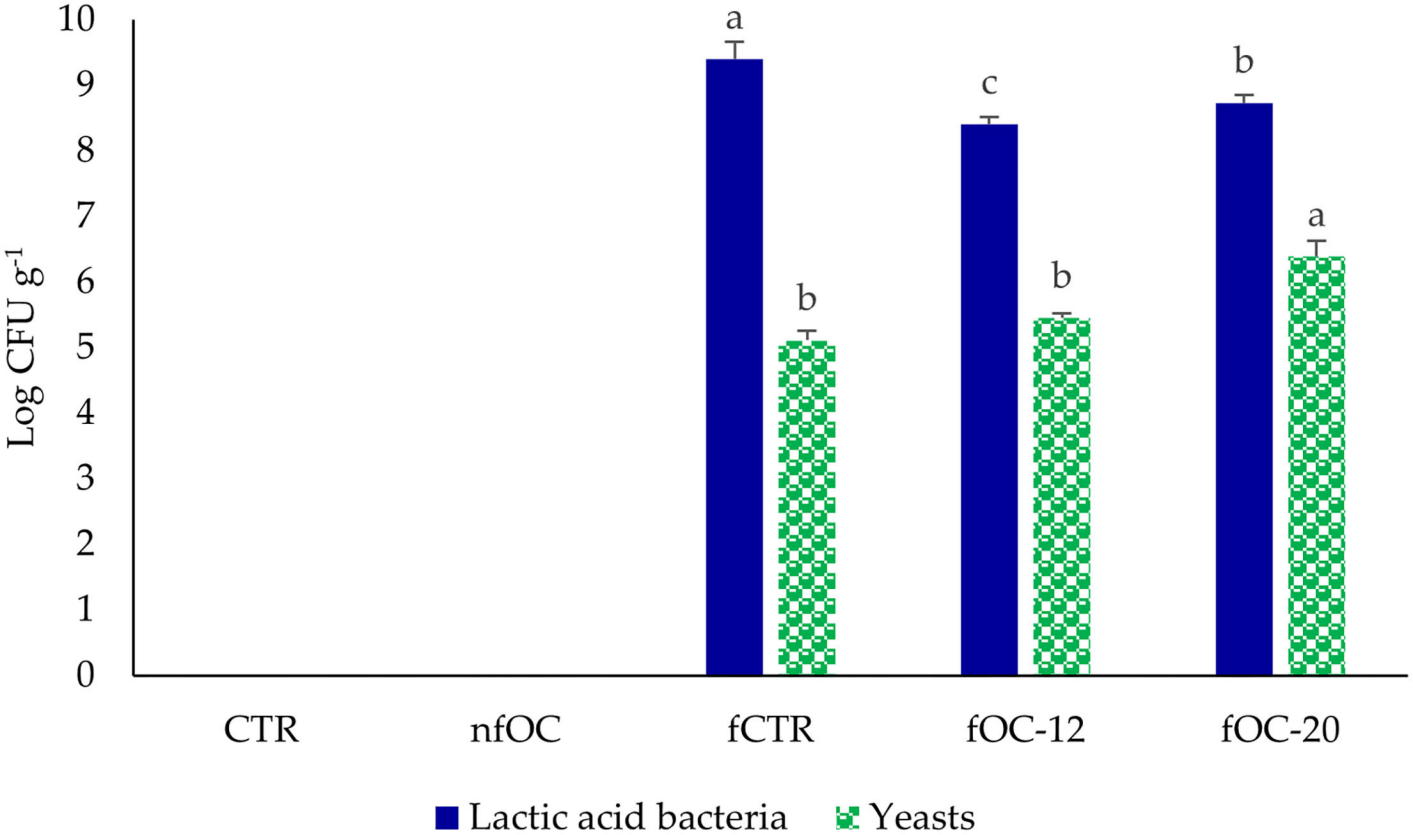
Cell density (log CFU g^−1^) of presumptive lactic acid bacteria and yeasts in gluten-free breadstick doughs. CTR, control breadsticks obtained by the dough without OC addition and without microbial fermentation; fCTR, breadsticks obtained by the dough without OC addition and fermented; nfOC, breadsticks obtained by the dough supplementation with 1% OC without microbial fermentation; fOC-12, breadsticks obtained by the dough supplementation with 1% OC and fermented for 12 h; fOC-20, breadsticks obtained by the dough supplementation with 1% OC and fermented for 20 h. The values represent means of triplicates; different letters indicate significant differences (*p* < 0.05) according to Tukey's Honestly Significant Difference (HSD) test.

The strains of *L. plantarum, L. plantarum/P. pentosaceus* and *K. marxianus*, which demonstrated to well adapt to OC matrix that was rich in polyphenols, were used as a mixed starter to ferment GF dough. In agreement with traditional sourdoughs, which showed that the ratio of cell counts of yeasts to lactobacilli ranges from 1:10 to 1:100 (40), in this work, we used the ratio of 1:100. The acidification curve of the mixed starter during preliminary dough fermentation at 30°C shows that the 4.6 pH value was reached after 20 and 12 h of fermentation with and without the addition of 10 g L^−1^ of OC, respectively. Therefore, these fermentation times were adopted in subsequent fCTR (20 h) and fOC (12 and 20 h, referred to as fOC-12 and fOC-20) experiments. Fermented GF breadstick doughs were thus prepared under these conditions and compared to unfermented ones with and without OC addition (CTR and nfOC). Changes in LAB and yeast cell densities and acidification were monitored in all doughs before and after driven fermentation (Figure 2). LAB and yeasts profiles changed as a consequence of OC addition and/or microbial fermentation. Before fermentation, all fermented doughs were characterized for ca. 7 and 5 log CFU g^−1^ for LAB and yeast microbial densities, respectively, corresponding to the initial inoculum of the starter, while cell densities were <1 log CFU g^−1^ in non-inoculated doughs. In fOC samples, the final cell count of presumptive LAB after 12 and 20 h of fermentation at 30°C was ~8.41 ± 0.1 and 8.73 ± 0.12 log CFU g^−1^. Yeast number increased up to 1 log cycle in fOC-20 dough. As already reported, sourdough originates from the starter culture-driven or spontaneous fermentation of a blend of flour and water, and in the first step, redox potential decreases favoring the growth of LAB (13). The pH values of doughs varied between 6.48 (CTR) and 6.00 (in dough with OC before fermentation). In fermented doughs, after 20 h, the lower pH was observed in fOC-20 (4.54). Previous studies already reported the ability of LAB, especially *L. plantarum*, to grow in the presence of relatively high polyphenol concentrations. Particularly, *L. plantarum* was able to colonize olives and its derivates, as shown by Blana et al. (41), in agreement with a previous work performed in our laboratory on bacterial microbiota on the surface of table olives fermented with the same commercial *L. plantarum* strain (21). Not only *Lactiplantibacillus* strains, but also *Pediococcus* strains should be adapted to the environment of the fermentation of olive products. As reported by Bonatsou et al. (42), *Pediococcus* strains are able to ferment olive products and decrease the pH value through the production of specific organic acids in the presence of phenolic compounds.

### Chemical and Nutritional Characterization

Gluten-free (GF) breadsticks were characterized by proximate composition, showing that the final moisture in the samples was significantly higher in the samples subjected to the fermentation process, reaching the highest value in fCTR (5.83 g 100 g^−1^). The highest fat content was found in nfOC due to the contribution of lipids from OC. However, in the fermented samples added with OC, the fat content was lower than the other samples, probably due to a loss of lipids that was observed during cooking. In fact, fermentation can affect the molecular structure of starch with subsequent modifications in dough rheology (43). Slight differences were found between the samples for proteins. The dietary fiber content was higher than 3 g 100 g^−1^, allowing to label the product with the claim “rich in fiber,” due the use of ingredients, such as inulin, *psyllium* and OC even if the samples added with OC did not show a significantly higher content in fiber than the CTR samples (Table 3).

**Table 3:** Proximate composition and antioxidant activity of breadsticks.

**Parameters**	**CTR**	**fCTR**	**nfOC**	**fOC-12**	**fOC-20**
Moisture (g 100 g^−1^)	2.65 ± 0.08c	5.83 ± 0.05a	2.99 ± 0.05c	5.02 ± 0.08b	5.08 ± 0.51b
Fats (g 100 g^−1^)	9.48 ± 0.02b	9.53 ± 0.40b	11.11 ± 0.03a	7.40 ± 0.04c	7.78 ± 0.90c
Proteins (g 100 g^−1^)	6.25 ± 0.20a	6.14 ± 0.04ab	6.17 ± 0.10ab	6.18 ± 0.02ab	6.05 ± 0.01b
Dietary fibers (g 100 g^−1^)	3.85 ± 0.98a	4.23 ± 0.66a	4.55 ± 0.75a	4.12 ± 0.86a	3.95 ± 0.99a
Ashes (g 100 g^−1^)	2.28 ± 0.01b	2.33 ± 0.01c	2.05 ± 0.01d	2.43 ± 0.01a	2.44 ± 0.02a
Carbohydrates (g 100 g^−1^)	79.34	76.17	77.68	78.97	78.65
TPC (μg g^−1^)	156 ± 2e	427 ± 15d	558 ± 17c	705 ± 5b	998 ± 5a
DPPH (μmol TE kg^−1^)	838 ± 12e	1,571 ± 27d	2,212 ± 18c	2,261 ± 8b	2,925 ± 25a
ABTS (μmol TE kg^−1^)	706 ± 13e	1,427 ± 6d	1,734 ± 18c	2,003 ± 11b	2,587 ± 18a

*The values are expressed as means of triplicates ± standard deviation; means followed by different letters in the same row are significantly different (p < 0.05) according to one-way ANOVA and Tukey's HSD test. ABTS, 2,2-Azino-bis(3-ethylbenzothiazoline-6-sulphonic acid; DPPH, 2,2-diphenyl-1-picrylhydrazyl; TE, Trolox equivalents; TPC, total phenol content; CTR, control breadsticks obtained by the dough without OC addition and without microbial fermentation; fCTR, breadsticks obtained by the dough without OC addition and fermented; nfOC, breadsticks obtained by the dough supplementation with 1% OC without microbial fermentation; fOC-12, breadsticks obtained by the dough supplementation with 1% OC and fermented for 12 h; fOC-20, breadsticks obtained by the dough supplementation with 1% OC and fermented for 20 h*.

As reported in Table 3, OC is a source of different bioactive compounds, such as phenolic compounds and tocopherols. Antioxidant activities of two different fermented GF breadstick doughs fortified with OC were investigated and compared to a non-inoculated GF breadstick dough added with OC and to two control GF breadstick doughs that were without OC-fermented and not fermented, respectively. The total phenol content (TPC) and the antioxidant activity of the GF breadsticks are reported in Table 3. Both OC addition and fermentation improved TPC and antioxidant activity of breadsticks. Fermentations may lead to significant changes to the health-promoting features of plant foods. Cereals *per se* contain classes of phytochemicals, but processing conditions markedly influence their levels and bioavailability (44). Among processing conditions, the sourdough fermentation has the most pronounced impact on the levels and bioavailability of phytochemicals. Those can be attributed to the capacity of sourdough fermentation to increase the levels of extractable phenolic compounds (13) which, in turn, were responsible for the enhancement of the antioxidant capacity, as measured by 2,2′-azino-bis(3-ethylbenzothiazoline-6-sulfonic acid) (ABTS) and DPPH assays. Difonzo et al. (26) showed how olive leaves polyphenols increased the antioxidant activity of baked salty snacks. Moreover, despite the loss of phenolic compounds during kneading and cooking phases ranging from 20 to 47%, different authors found that olive phenolics are able to reduce the oxidation degree in baked products and bring health-related benefits by lowering the postprandial oxidized low-density lipoproteins (45, 46). The incorporation of extracts from olive leaves and wastewater also resulted in improved nutritional and functional properties of GF breadsticks, as evidenced by the changes observed in the insoluble/soluble polyphenol ratio in favor of the soluble fraction, the enhanced bioavailability of polyphenols, and the higher antioxidant activity (47).

### *In vitro* Starch Hydrolysis

**Figure 3:**
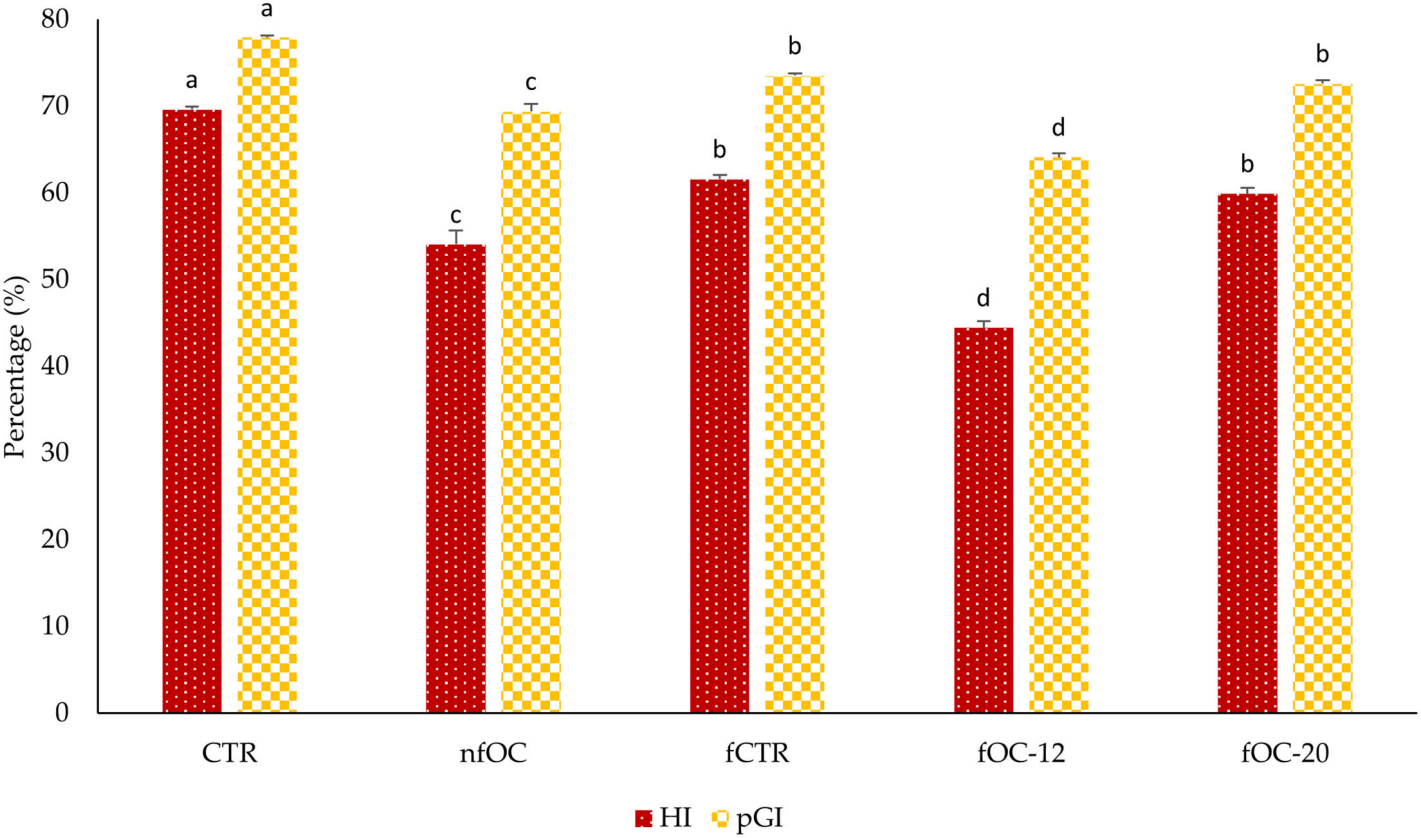
Results of hydrolysis index (HI) and predicted glycemic index (pGI). CTR, control breadsticks obtained by the dough without OC addition and without microbial fermentation; fCTR, breadsticks obtained by the dough without OC addition and fermented; nfOC, breadsticks obtained by the dough supplementation with 1% OC without microbial fermentation; fOC-12, breadsticks obtained by the dough supplementation with 1% OC and fermented for 12 h; fOC-20, breadsticks obtained by the dough supplementation with 1% OC and fermented for 20 h. The values represent means of triplicates; different letters indicate significant differences (*p* < 0.05) according to Tukey's HSD test.

As shown in Figure 3, after 12 h of fermentation (fOC-12), pGI and HI changed significantly (*p* < 0.05) compared to CTR. In fact, the lowest value of pGI was found in fOC-12 at approximately 64.11 ± 0.42. CTR has a statistically higher pGI value (77.94) than other samples. The HI and pGI values of samples with OC (fOC-12 and nfOC) were statistically lower than samples without OC (fCTR). However, samples fermented without OC also showed statistically lower pGI values than CTR.

It is well recognized that fermentation reduced pGI. Taking an example of bread, consumption of sourdough bread reduced postprandial blood glucose and insulin response. This mechanism could explain why organic acids, such as lactic and acetic acids, produced in sourdough lower its estimated pGI. Acetic acid appears to be associated with a delay in gastric emptying, whereas lactic acid induces interactions between starch and gluten during dough baking and reduces starch availability (48). These results were in accordance with Curiel et al. (49). In fact, the biological acidification of microorganisms is one of the main factors that decrease starch hydrolysis rate and HI. Moreover, wheat bread fortified with sourdough-fermented hemp flour showed a lower pGI compared to bread fermented with baker's yeast (50).

### Colorimetric and Textural Evaluation

As shown in Table 4, the addition of OC led to an L^*^ decrease due to the darkening of breadsticks; this effect is due to the dark color of OC and could be related to the presence of sugars. In fact, Kocadagli et al. (51) found that the concentration of reducing sugars in cookies increased significantly during baking and contributed to browning through caramelization, suggesting that reducing sugars from OC may contribute to browning. The same trend was found for the yellowness index b^*^ that was the highest in fCTR, whereas the redness index a^*^ increased with OC addition.

**Table 4:** Color and texture profile analysis of gluten-free breadsticks.

		**CTR**	**fCTR**	**nfOC**	**fOC-12**	**fOC-20**
**Colorimetric parameters**	L*	66.06 ± 0.29a	59.60 ± 0.23b	49.73 ± 0.32c	46.12 ± 0.18d	44.85 ± 0.86e
	b*	31.50 ± 0.72b	33.56 ± 0.35a	27.96 ± 0.72c	28.85 ± 0.34c	27.45 ± 0.43c
	a*	7.06 ± 0.41d	11.24 ± 0.29b	7.84 ± 0.14c	10.98 ± 0.27b	12.56 ± 0.17a
**Texture parameters**	Hardness (N)	16.62 ± 1.96c	37.04 ± 0.80b	15.04 ± 0.48c	53.73 ± 0.56a	52.04 ± 3.21a
	Brittleness (mm)	0.043 ± 0.001d	0.106 ± 0.001b	0.032 ± 0.004d	0.158 ± 0.006a	0.090 ± 0.005c

*The values are expressed as means of triplicates ± standard deviation; means followed by different letters in the same row are significantly different (p < 0.05) according to Tukey's Honestly Significant Difference (HSD) test. CTR, control breadsticks obtained by the dough without OC addition and without microbial fermentation; fCTR, breadsticks obtained by the dough without OC addition and fermented; nfOC, breadsticks obtained by the dough supplementation with 1% OC without microbial fermentation; fOC-12, breadsticks obtained by the dough supplementation with 1% OC and fermented for 12 h; fOC-20, breadsticks obtained by the dough supplementation with 1% OC and fermented for 20 h*.

The texture is one of the most critical quality attributes in breadsticks because consumers appreciate a crisp and crunchy texture (52). From a textural point of view, baked snacks like breadsticks are characterized by a rigid, stiff structure with a little tendency to deform before fracture when subjected to small forces (47). In the present study, the different mechanical behavior of the experimental breadsticks was measured on the day of baking to assess their quality in terms of hardness and brittleness. As reported in Table 4, both CTR and nfOC significantly (*p* < 0.05) lowered the maximum force needed to break the experimental breadsticks. In particular, while the control sample and the samples added with OC showed the lowest values of force at break, the most pronounced increase in hardness values was observed in the samples subjected to dough fermentation. Brittleness, which is a textural parameter describing the distance crossed by the blade through the sample before its breaking and thus, how far a sample can be deformed before fracture, showed the lowest values in CTR and nfOC according to the results from hardness. In fact, the higher the brittleness value, the less friable is the product. Texture profile analysis had been created as an imitative test that resembles what goes on in the human mouth and is a parameter to determine the human perception of the texture of the product and how it behaved when handled and eaten. Furthermore, it incorporates all the attributes (mechanical, geometric and surface) of the food. In fact, physical texture is also an important quality to determine the porous structure of baked products where the texture is related to the geometric and mechanical properties of the product, which heavily depends on its cellular structure, such as cell wall thickness, cell size and uniformity (53). A possible explanation for this hardening effect could be related to the loss of lipids during cooking of fOC-12 and fOC-20. Therefore, the use of leavening agent, as sourdough, may drive changes in product structure, altering water-holding capacity of the products which can significantly affect oil uptake capacity of the products, as observed by Marquez et al. (54). This evaluates the possibility to obtain taralli biscuits with limited water absorption during storage and consequently, prolonged hardness and crispness characteristics. In addition, during the baking stage in the oven, the water vapor carries fats/oils emulsified with it toward the external layer. While the vapor leaves the structure, the oil stays on the surface. Moreover, sourdough fermentation is known to cause transformations of lipids and macromolecules (40, 55). Oxidation of components, such as lipids and proteins (including protein carbonylation), as well as the interactions between raw material components (e.g., protein-lipid or protein-protein interactions), take place during fermentation (56). Based on this consideration, differences between breadsticks obtained by baking powder only or sourdough technology on fat loss may be influenced by different structural components, such as changes in the pH value of the dough system which, in turn, affects this phenomenon.

### Sensory Evaluation and Aromatic Profile

**Figure 4:**
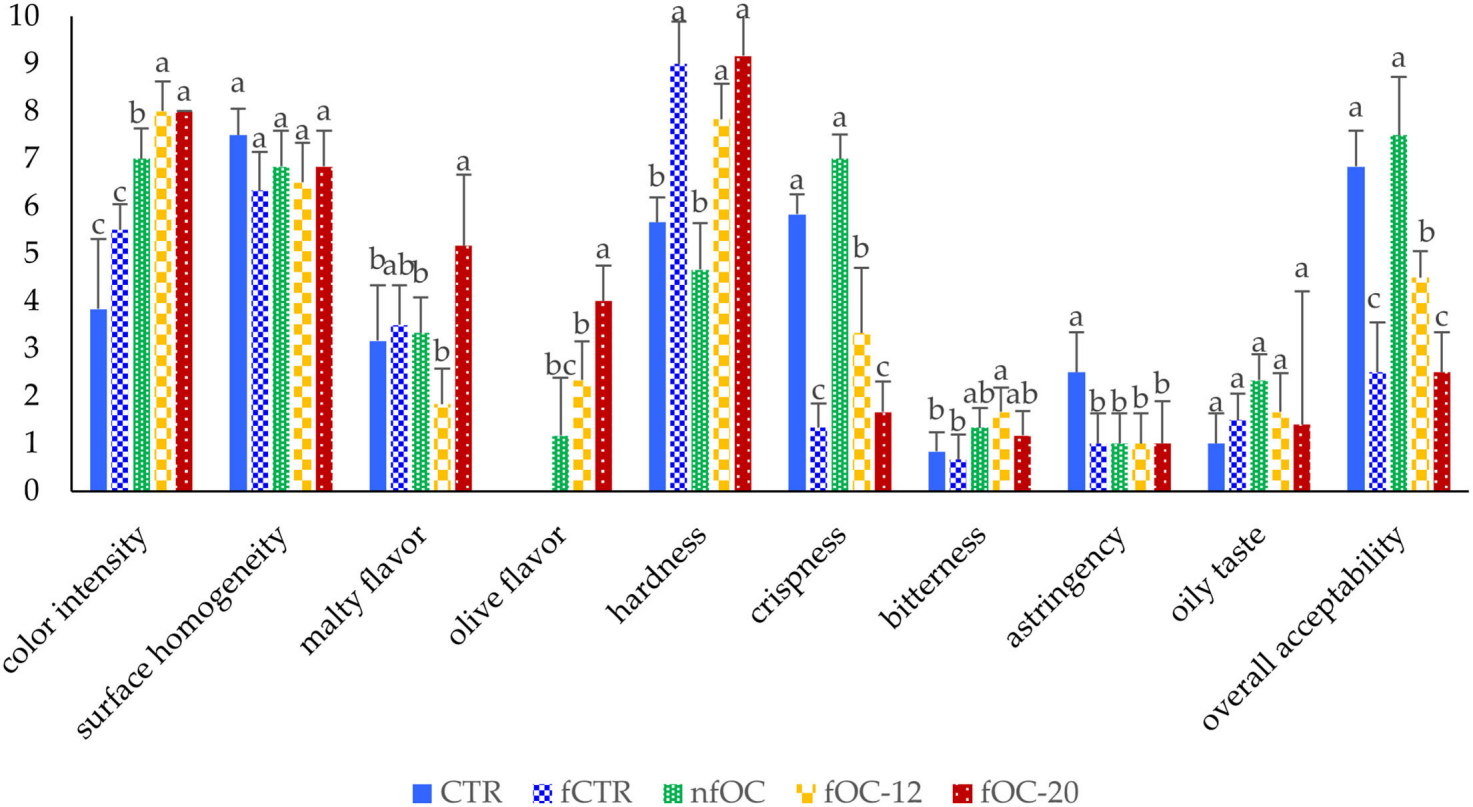
Sensory evaluation of the gluten-free breadsticks. Different letters indicate significantly different (*p* < 0.05) according to one-way ANOVA and Tukey's HSD test. CTR, control breadsticks obtained by the dough without OC addition and without microbial fermentation; fCTR, breadsticks obtained by the dough without OC addition and fermented; nfOC, breadsticks obtained by the dough supplementation with 1% OC without microbial fermentation; fOC-12, breadsticks obtained by the dough supplementation with 1% OC and fermented for 12 h; fOC-20, breadsticks obtained by the dough supplementation with 1% OC and fermented for 20 h.

Some of the selected sensory attributes were influenced by the OC addition and fermentation (*p* < 0.05) as reported in Figure 4. In novel food product formulation, maintaining the sensory characteristics of the product is of great importance. The fermentation led to a worsening of textural attributes. In fact, all the fermented samples had the higher scores in hardness and the lowest in crispness according to the results obtained from the texture profile analysis (Table 4). However, CTR and the samples added with OC without dough fermentation led to the highest scores in crispness and overall acceptability. The addition of OC did not induce an astringency and bitterness perception, except for fOC-12 that was perceived as the more bitter. The results of overall acceptability were completely in line with the results of hardness and crispness, highlighting that these parameters affected the whole liking of the breadsticks.

Fermentation is an important biotechnology strategy for maintaining and/or improving safety, nutritional, sensory and shelf-life properties of foods (33). Composition and concentration of volatile flavor-aroma compounds have been of great interest because of their important influence on sensory properties and consumer acceptance. Although, most of the volatile compounds are naturally present in the raw materials, it does not neglect that they might be synthesized as secondary metabolites by microbes (57). Considering the potential unpleasant aromatic properties of OC, lactic acid fermentation through selected strains may be considered as an interesting option to change the flavor profile of processed GF foods added to this by-product and ensure a better control of flavor changes during processing. In this framework, some authors have evaluated how different formulations and processing may affect the volatile and lipidic fraction of taralli (58–60). Table 5 reports the main volatile compounds detected in the samples. Several pyrazine derivatives, such as dimethyl-pyrazines or ethyl-methyl-pyrazines, whose origin could be linked to the Maillard reaction. Besides nitrogen-containing compounds, the Maillard reaction also arise several furans that were also detected in the experimental samples. Pico et al. (61, 62), in studying the aromatic profile of several GF breads obtained from flours and starches of different origins, selected a list of the main volatiles identified in all GF samples, including compounds deriving from fermentation process, lipid oxidation and Strecker degradation. In this framework, 2-methylbutanal and 3-methylbutanal, Strecker aldehydes deriving from Maillard reaction, were more present in the volatile pattern of control breadsticks followed by the samples added with OC. A similar trend was found for all the aldehydes, including the aldehydes from lipid oxidation with the lowest concentration in the fermented samples, irrespective of the OC addition. On the contrary, the amount of benzaldehyde was the highest in the samples added with OC since it is a compound generally founded in *Drupaceae* (63). 2-Butanone was already detected in baked goods in previous researches and its origin was attributed to β-chetoacids, which arose from thermal treatment of tryglicerides *via* decarboxylation (64). The furan compounds were mostly represented by furfural, a caramel-like odorant derived by Maillard reaction, which is significantly more abundant in acorn-added biscuits than in control. Furfural is typically present in biscuits as reported in several studies (65–68). Pyrazines, produced by Maillard reaction, were also detected. In most cases, the concentration of all these volatile compounds was reduced by dough fermentation. This behavior could be associated to the variation of the matrix composition during kneading and baking of the fermented samples, leading to modifications of the structure, viscosity and of physicochemical interactions established between volatile and non-volatile compounds (69). Moreover, lipid fraction also influences the release of volatile compounds in food matrices (70).

**Table 5:** Volatile compounds (μg g^−1^) detected in the different samples of breadsticks.

**Volatile compounds**	
**Aldehydes**	**CTR**	**fCTR**	**nfOC**	**fOC-12**	**fOC-20**
Propanal 2-methyl	12.65 ± 1.02a	1.33 ± 0.14c	10.85 ± 0.41b	2.50 ± 0.32c	1.45 ± 0.26c
Butanal, 2-methyl-	36.27 ± 1.75a	2.27 ± 0.27c	29.41 ± 0.70b	1.35 ± 0.10c	2.77 ± 0.17c
Butanal, 3-methyl-	82.63 ± 5.41a	12.21 ± 0.92c	59.90 ± 0.74b	8.64 ± 0.64c	7.38 ± 0.41c
Pentanal	5.57 ± 1.11a	2.27 ± 0.24b	5.17 ± 0.23a	2.09 ± 0.06b	2.07 ± 0.16b
2-Butenal	1.14 ± 0.57a	0.39 ± 0.04b	0.60 ± 0.08ab	0.19 ± 0.01b	0.15 ± 0.03b
Hexanal	39.11 ± 8.81a	25.56 ± 2.23bc	29.51 ± 1.35ab	15.17 ± 0.41cd	12.98 ± 0.41d
Octanal	1.56 ± 0.46a	0.73 ± 0.17a	1.77 ± 0.13a	1.17 ± 0.62a	1.17 ± 0.62a
Nonanal	12.93 ± 3.72a	5.67 ± 0.47b	12.38 ± 2.12a	6.94 ± 0.37b	4.41 ± 0.22b
*trans*-2-hexenal	nd	nd	1.30 ± 0.32a	0.85 ± 0.14ab	0.75 ± 0.11b
*trans*-2-octenal	1.33 ± 0.27b	1.12 ± 0.19b	2.46 ± 0.11a	1.05 ± 0.07bc	0.68 ± 0.08c
Benzaldehyde	3.04 ± 1.34d	2.09 ± 0.27d	73.05 ± 5.52a	31.67 ± 2.53b	21.28 ± 0.97c
**Ketons and esters**	
2-Butanone	3.89 ± 0.20b	nd	13.14 ± 0.58a	1.99 ± 0.14c	2.12 ± 0.18c
2-Pentanone	0.53 ± 0.09b	0.09 ± 0.01c	0.70 ± 0.06a	0.17 ± 0.02c	0.10 ± 0.01c
2,3-Butanedione	19.42 ± 1.77a	8.17 ± 1.21b	19.14 ± 0.77a	9.57 ± 0.33b	7.41 ± 0.41b
2,3-Pentanedione	26.00 ± 6.48a	8.86 ± 1.05b	25.65 ± 1.77a	8.72 ± 0.60b	7.19 ± 0.26b
1-Octen-3-one	0.62 ± 0.16b	0.41 ± 0.01b	0.86 ± 0.09b	1.48 ± 0.31a	1.43 ± 0.25a
Ethyl acetate	nd	3.18 ± 0.10b	nd	3.88 ± 0.56b	11.74 ± 1.19a
**Acids**	
Acetic acid	3.68 ± 0.98a	3.35 ± 0.59a	1.35 ± 0.15b	0.77 ± 0.15b	1.18 ± 0.14b
**Furans**					
Furan	3.82 ± 0.48a	1.10 ± 0.16c	2.82 ± 0.29ab	2.02 ± 0.51bc	2.10 ± 0.46bc
Furan, 2-methyl-	12.78 ± 0.90a	5.66 ± 0.33b	12.88 ± 1.06a	6.62 ± 1.27b	8.20 ± 1.03b
2-Furancarboxaldehyde	11.03 ± 3.08a	8.34 ± 1.08a	10.86 ± 0.51a	7.87 ± 0.74a	7.11 ± 0.86a
2-Furanmethanol	38.57 ± 2.24a	4.24 ± 0.12d	30.60 ± 2.48b	16.40 ± 1.94c	6.66 ± 1.44d
**Pyrazines**	
Pyrazine	6.36 ± 0.67a	nd	6.13 ± 0.39a	0.20 ± 0.01b	nd
Pyrazine, methyl-	36.40 ± 2.50a	1.61 ± 0.43b	38.71 ± 0.70a	4.52 ± 0.46b	nd
Pyrazine, 2,5-dimethyl-	17.21 ± 1.13a	1.98 ± 0.49c	12.30 ± 1.34b	nd	nd
Pyrazine, ethyl-	17.16 ± 1.41ª	2.91 ± 0.61bc	19.27 ± 1.59a	4.13 ± 0.38b	0.47 ± 0.14c
Pyrazine, 2,3-dimethyl-	9.58 ± 0.65ª	0.44 ± 0.09c	5.87 ± 0.18b	0.43 ± 0.01c	nd
Pyrazine, 2-ethyl-5-methyl-	9.10 ± 0.94b	1.70 ± 0.85c	13.22 ± 1.38a	1.14 ± 0.50c	0.32 ± 0.01c

*The values are expressed as means of triplicates ± standard deviation; means followed by different letters in the same row are significantly different (p < 0.05) according to one-way ANOVA and Tukey's HSD test. CTR, control breadsticks obtained by the dough without OC addition and without microbial fermentation; fCTR, breadsticks obtained by the dough without OC addition and fermented; nfOC, breadsticks obtained by the dough supplementation with 1% OC without microbial fermentation; fOC-12, breadsticks obtained by the dough supplementation with 1% OC and fermented for 12 h; fOC-20, breadsticks obtained by the dough supplementation with 1% OC and fermented for 20 h*.

## Conclusion

This study aimed to use two selected lactic acid bacteria and a yeast for increasing the antioxidant features and the phenol profile of GF breadsticks fortified with OC with the perspective of producing a functional food. Our results showed that both OC addition and fermentation improved TPC and antioxidant activity of breadsticks. Furthermore, the pGI was influenced by OC addition and dough fermentation. In fact, the lowest value of pGI was found in fOC-12, and HI and pGI values of samples with OC (fOC-12 and nfOC) were statistically lower than samples without OC (fCTR). The samples subjected to dough fermentation showed the highest hardness values, as evidenced from both texture profile analysis and sensory evaluation. Moreover, in most cases, the concentration of the detected volatile compounds was reduced by fermentation. Additional trials are necessary to improve the textural and sensorial features of the GF breadsticks.

In conclusion, the fermentation of agri-food residues by LAB, alone or in combination with other microorganisms, opens an avenue of opportunities for a sustainable circular economy. In fact, our work highlights the potential of OC to be exploited by fermentation to produce GF-baked goods with an improved nutritional profile.

## Data Availability Statement

The raw data supporting the conclusions of this article will be made available by the authors, without undue reservation.

## Author Contributions

GD and MDA dealt with conceptualization. GC, GD, GdG, and MC performed analysis, data elaboration, and the first drafting of the article. GC, GD, MC, and AP supervised the final revision of the manuscript. All authors approved the final version of the manuscript to be submitted for publication.

## Funding

This work was supported by POR Puglia 2014/2020-Asse X-Azione 10.4 Research for Innovation-REFIN Code No. E65BAEEE.

## Conflict of Interest

The authors declare that the research was conducted in the absence of any commercial or financial relationships that could be construed as a potential conflict of interest.

## Publisher's Note

All claims expressed in this article are solely those of the authors and do not necessarily represent those of their affiliated organizations, or those of the publisher, the editors and the reviewers. Any product that may be evaluated in this article, or claim that may be made by its manufacturer, is not guaranteed or endorsed by the publisher.
