# Supplementary material for: Nutritional Improvement of Gluten-Free Breadsticks by Olive Cake Addition and Sourdough Fermentation: How Texture, Sensory, and Aromatic Profile Were Affected?
Source: Front Nutr. 2022 Feb 10;9:830932. doi: 10.3389/fnut.2022.830932 (PMC8869757; doi:10.3389/fnut.2022.830932)
Supplement: Supplementary file 1 [file Table_1.docx]

Supplementary material

| **Table S1.** pH and cell density (log CFU g^−1^) of selected lactic acid bacteria and yeasts strains after growth for 24 h at 30 or 25 °C in de Man Rogosa and Sharpe or Yeast Extract Peptone Dextrose (YPD) media respectively added of different concentration (1, 2, 5, 10, 20 g L^-1^) of olive cake (OC). The data are an average of three independent experiments ± the standard deviation. | | | | | | | | | | | | |
| --- | --- | --- | --- | --- | --- | --- | --- | --- | --- | --- | --- | --- |
| **Strain** | **OC concentration (g L^−1^)** | | | | | | | | | | | |
|  | **Control (no OC)** | | **1 g L^−1^** | | **2 g L^−1^** | | **5 g L^−1^** | | **10 g L^−1^** | | **20 g L^−1^** | |
|  | **pH** | **log CFU g^-1^** | **pH** | **log CFU g^-1^** | **pH** | **log CFU g^-1^** | **pH** | **log CFU g^-1^** | **pH** | **log CFU g^-1^** | **pH** | **log CFU g^-1^** |
| *Hanseniaspora uvarum* CNG11 | 5.85 ± 0.19 | 8.95 ± 0.23 | 5.58 ± 0.02 | 8.87 ± 0.24 | 5.65 ± 0.01 | 8.55 ± 0.11 | 6.02 ± 0.05 | 7.23 ± 0.03 | 6.06 ± 0.05 | 6.92 ± 0.07 | 5.92 ± 0.19 | 6.65 ± 0.06 |
| *Kluyveromyces marxianus* KL | 5.75 ± 0.07 | 9.44 ± 0.12 | 5.51 ± 0.05 | 9.25 ± 0.07 | 5.38 ± 0.09 | 9.05 ± 0.08 | 5.39 ± 0.03 | 8.72 ± 0.12 | 5.41 ± 0.11 | 8.59 ± 0.11 | 5.95 ± 0.18 | 6.9 ± 0.02 |
| *Lactiplantibacillus plantarum* V3-D0001 | 3.34 ± 0.11 | 9.51 ± 0.28 | 3.32 ± 0.01 | 9.45 ± 0.11 | 3.69 ± 0.04 | 9.22 ± 0.15 | 4.36 ± 0.06 | 8.78 ± 0.07 | 4.46 ± 0.02 | 8.50 ± 0.01 | 5.97 ± 0.13 | 7.35 ± 0.09 |
| *L. plantarum/Pediococcus pentosaceus* CBD 100-D0001 | 3.35 ± 0.16 | 9.37 ± 0.26 | 3.31 ± 0.03 | 9.38 ± 0.17 | 3.82 ± 0.09 | 9.11 ± 0.12 | 4.32 ± 0.01 | 8.68 ± 0.05 | 4.55 ± 0.04 | 8.55 ± 0.05 | 6.01 ± 0.21 | 7.21 ± 0.00 |
| *Leuconostoc mesenteroides* DDL1 | 3.45 ± 0.03 | 9.21 ± 0.14 | 3.38 ± 0.03 | 9.15 ± 0.26 | 3.78 ± 0.04 | 8.98 ± 0.09 | 4.79 ± 0.07 | 8.25 ± 0.05 | 5.02 ± 0.03 | 7.98 ± 0.06 | 6.01 ± 0.24 | 7.15 ± 0.04 |
| *Lc. mesenteroides* KI6 | 3.63 ± 0.18 | 9.15 ± 0.25 | 3.33 ± 0.01 | 9.09 ± 0.26 | 3.76 ± 0.06 | 8.84 ± 0.07 | 4.8 ± 0.02 | 8.09 ± 0.09 | 6.06 ± 0.04 | 7.74 ± 0.10 | 5.99 ± 0.15 | 7.04 ± 0.15 |
| *Pichia kudriavzevii* DCNa1 | 5.94 ± 0.22 | 8.88 ± 0.22 | 5.65 ± 0.05 | 8.85 ± 0.14 | 5.18 ± 0.08 | 8.62 ± 0.07 | 5.59 ± 0.09 | 8.00 ± 0.08 | 5.84 ± 0.10 | 7.56 ± 0.09 | 5.98 ± 0.20 | 6.35 ± 0.15 |
| *Saccharomyces cerevisiae* DDNd10 | 5.93 ± 0.23 | 9.11 ± 0.11 | 5.68 ± 0.05 | 9.05 ± 0.35 | 5.23 ± 0.06 | 8.77 ± 0.12 | 5.54 ± 0.10 | 7.63 ± 0.09 | 5.96 ± 0.05 | 7.32 ± 0.08 | 5.95 ± 0.19 | 6.76 ± 0.07 |
| *Saccharomyces subpellicelosus* DFNb1 | 5.87 ± 0.19 | 9.01 ± 0.21 | 5.62 ± 0.01 | 8.95 ± 0.35 | 5.19 ± 0.03 | 8.82 ± 0.12 | 5.78 ± 0.11 | 7.76 ± 0.064 | 6.00 ± 0.15 | 7.18 ± 0.13 | 5.97 ± 0.22 | 6.78 ± 0.02 |
